# A genomic perspective on a new bacterial genus and species from the *Alcaligenaceae* family, *Basilea psittacipulmonis*

**DOI:** 10.1186/1471-2164-15-169

**Published:** 2014-03-01

**Authors:** Katrine L Whiteson, David Hernandez, Vladimir Lazarevic, Nadia Gaia, Laurent Farinelli, Patrice François, Paola Pilo, Joachim Frey, Jacques Schrenzel

**Affiliations:** 1 Genomic Research Laboratory, Department of Internal Medicine, Service of Infectious Diseases, Geneva University Hospitals, Gabrielle-Perret-Gentil 4, CH-1211 Geneva 14, Switzerland; 2 Fasteris SA, CH-1228 Plan-les-Ouates, Switzerland; 3 Institute of Veterinary Bacteriology, University of Bern, Länggassstrasse 122, Postfach, CH-3001 Bern, Switzerland; 4 Clinical Microbiology Laboratory, Department of Internal Medicine, Service of Infectious Diseases, Geneva University Hospitals, Gabrielle-Perret-Gentil 4, CH-1211 Geneva 14, Switzerland; 5 Current address: Department of Biology, San Diego State University, San Diego, CA, USA

**Keywords:** Bacteria, Parakeet, High-throughput sequencing, Genome, Phylogenetic profile

## Abstract

**Background:**

A novel Gram-negative, non-haemolytic, non-motile, rod-shaped bacterium was discovered in the lungs of a dead parakeet (*Melopsittacus undulatus*) that was kept in captivity in a petshop in Basel, Switzerland. The organism is described with a chemotaxonomic profile and the nearly complete genome sequence obtained through the assembly of short sequence reads.

**Results:**

Genome sequence analysis and characterization of respiratory quinones, fatty acids, polar lipids, and biochemical phenotype is presented here. Comparison of gene sequences revealed that the most similar species is *Pelistega europaea*, with BLAST identities of only 93% to the 16S rDNA gene, 76% identity to the *rpoB* gene, and a similar GC content (~43%) as the organism isolated from the parakeet, DSM 24701 (40%). The closest full genome sequences are those of *Bordetella* spp. and *Taylorella* spp. High-throughput sequencing reads from the Illumina-Solexa platform were assembled with the Edena *de novo* assembler to form 195 contigs comprising the ~2 Mb genome. Genome annotation with RAST, construction of phylogenetic trees with the 16S rDNA (*rrs*) gene sequence and the *rpoB* gene, and phylogenetic placement using other highly conserved marker genes with ML Tree all suggest that the bacterial species belongs to the *Alcaligenaceae* family. Analysis of samples from cages with healthy parakeets suggested that the newly discovered bacterial species is not widespread in parakeet living quarters.

**Conclusions:**

Classification of this organism in the current taxonomy system requires the formation of a new genus and species. We designate the new genus *Basilea* and the new species *psittacipulmonis*. The type strain of *Basilea psittacipulmonis* is DSM 24701 (= CIP 110308 T, 16S rDNA gene sequence Genbank accession number JX412111 and GI 406042063).

## Background

The study of parakeet respiratory infection has had important implications for biomedical research since December of 1929, when psittacosis caused by *Chlamydophila psittaci* created a health scare which eventually led to the formation of the National Institutes of Health [1]. Here we describe a novel bacterium from the family *Alcaligenaceae* that was discovered in the lungs of a dead parakeet (*Melopsittacus undulatus*) from a petshop in Basel, Switzerland. The bacterial family *Alcaligenaceae* includes genera that have been isolated from humans, animals and the environment. They are Gram-negative rods or coccobacilli that possess oxidase and catalase, growing well on complex media under aerobic or microaerobic conditions.

There are nearly 25000 prokaryote genome projects registered in the NCBI database as of early 2014 [2], many of them human-associated. Pathogens of animals that are not important for agriculture or zoonotic transmission of disease are poorly studied. Filling out the tree of life is important for improving genome sequence annotation and creating good phylogenetic landmarks to analyze metagenomic data [3,4].

The genome of a bacterium isolated from the lungs of a parakeet (*Melopsittacus undulatus)* in captivity was sequenced using Illumina sequencing. Here we describe the success and limitation of a comparative genomics approach to studying this newly discovered bacterium. This bacterium is most closely related to *Pelistega europaea* according to a Ribosome Database Project (RDP) classifier assessment of the similarity of their 16S rDNA (*rrs*) gene [5,6], a stable and frequently used phylogenetic marker [7]. The closest fully sequenced relatives, from genus *Taylorella* and genus *Bordetella*[8-11], share a great number of putative genes and functions, but are too distant to make specific analyses through simple sequence comparisons.

## Methods

### Bacterial isolation, phenotypic and biochemical characterization

The carcass of a suddenly dead parakeet (*M. undulatus*) from a petshop without previous presentation of clinical signs was brought to the Institute of Animal Pathology, University of Bern, Switzerland for post mortem examination and histological analysis.

Lung and liver samples from the deceased parakeet were cultured on tryptone soy agar with 5% sheep blood (Oxoid, Basel, Switzerland) at 37°C in an atmosphere of air with 5% CO_2_ for 48 hours_._ Phenotypic and biochemical characterization were performed with a VITEK2 instrument (bioMérieux, Geneva, Switzerland) and the API ZYM, API NH and API 20 NE (bioMérieux) according to the manufacturer’s instructions. Analysis of respiratory quinones, polar lipids and fatty acids were carried out by the Identification Service of the DSMZ and DR. BJ Tindall, DSMZ, Braunschweig, Germany. Plates were stained with 5% molybdophosphoric acid to show all lipids.

### Submission to international culture collections

The strain JF4266 was submitted to the Deutsche Sammlung von Mikroorganismen und Zellkulturen (DSMZ, deposited under the name *Alcaligenaceae* bacterium DSM 24701) and the Institute Pasteur (number CIP 110308 T) with the name *B. psittacipulmonis*. Both repositories have made the strain publicly available under the name *B. psittacipulmonis* in addition to the strain number assigned by each repository, in accordance with the Rules of Bacteriological Code (1990 revision) as revised by the International Committee on Systematics of Prokaryotes (ICSP) at the plenary sessions in Sydney and Paris [12].

### PCR conditions

The material from the bottom of three cages (with live parakeets) and cage water were obtained from three petshops in Switzerland and France. Cage water was concentrated 50-fold in a vacuum concentrator. The cage samples were mixed with the lysis buffer [final concentration Tris 10 mM, EDTA 1 mM (pH 8), Tween 0.5%, proteinase K (Fermentas, Burlington, Canada) 200 μg/ml] and incubated for 2.5 hours at 55°C [13]. Proteinase K was inactivated by a 10 min incubation at 95°C and the samples were frozen at -20°C. The PCR contained 6 μl of lysate and 0.5 μM of both forward and reverse primers in 50 μl of PrimeStar HS Premix (Takara, Otsu, Shiga, Japan). The PCR mix was amplified for 36 cycles (for three putative protein coding regions) or 30 cycles (for the 16S rDNA gene) of 98°C for 10 seconds, 56°C for 15 seconds, and 72°C for 1 min. One μl of the amplified reaction mix was run on the Agilent Bioanalyzer using a DNA1000 lab chip to determine if the product was generated. The Per-1 F/R, Per-2 F/R and Per-3 F/R primer pairs amplify 730, 522 and 533 bp regions of the DSM 24701 genomic DNA. The primers were designed to amplify RAST predicted genes of unknown function that are unique to the parakeet genome (there are no Blast hits to the nr/nt database). Primer pair Per-11 F/R specifically amplifies a unique298 bp region of the DSM 24701 16S rDNA, from position 202 to 499. Primer sequences were as follows: Per-1 F 5′ TCTGGGTGATTTTGGAGAGG 3′, Per-1R 5′ ATTCTCGCGTTCTTGCTGTT 3′, Per-2 F 5′ TTCGTATCTGGCAGAGGCTT 3′, Per-2R 5′ AACAATTGGGTTCCCACAAA 3′, Per-3 F 5′ AGATGATGGAGCAAGCTCGT 3′, Per-3R 5′ CAATTGGTCTACCGTTGCCT 3′, Per-11 F 5′ AAAGCAGGGGACCGCAAGGC 3′, Per-11R 5′ TCAGGTACCGTCATCACTCAATGGT 3′.

Controls to ensure that the parakeet cage samples did not inhibit PCR reactions were performed in two ways: 1) The parakeet cage material and water lysate were spiked with genomic DNA from DSM 24701*,* in which case all 3 pairs of DSM 24701 specific primers successfully amplified the expected product. 2) A PCR targeting the first three variable regions of the 16S rDNA gene (V123) was also performed on the parakeet cage samples using broad range bacterial 16S primers (8 F 5 GAGTTTGATCMTGGCTCAG 3 and 534R 5 CCGCGRCTGCTGGCAC 3). These primers amplified the expected segment of the bacterial 16S rDNA gene from all three parakeet cage material and water samples, suggesting that there are bacteria in the sample, as we would expect, but not DSM 24701*.*

### Sequencing

Genomic DNA was prepared using the procedure in Hernandez et al. [14] using the DNEasy kit (Qiagen, Venlo, Netherlands) and sequenced with the Solexa Illumina Genome Analyzer. The 454 sequencing was conducted by Microsynth in Balgach, Switzerland. Optical mapping was carried out by digestion of genomic DNA by *Nhe*I with OpGen in Madison, Wisconsin, USA.

### Assembly and annotation

The paired Illumina reads were assembled with the Edena assembler [14]. The assembly of 454 sequencing data was performed with the dedicated GS De Novo Assembler available from Roche (Roche Applied Science, Indianapolis, IN, USA). The final 195 contigs were submitted to the RAST server (Chicago, IL, USA) for annotation [15].

### Phylogenetic analysis

A 1535 bp segment of the 16S rDNA gene, found on contig 42 of the draft genome (Genbank accession number JX412111 and GI 406042063) was analyzed with the RDP Classifier [5]. Neighbor joining, maximum-parsimony and maximum-likelihood phylogenetic trees based on 16S rDNA sequence were constructed with MEGA 5 [16]. Similarly, a Neighbor Joining tree was constructed with the *rpoB* gene sequence from the draft genome, *Pelistega europaea*, and several related taxa. BLASTn was used to exhaustively search all 16S rDNA gene sequences available in the NCBI database (Table 1). The dinucleotide usage of the genomes was converted to a Bray-Curtis distance matrix and clustered using multidimensional scaling in Primer [17]. Clustered regularly interspaced Short Palindromic Repeats (CRISPR) detection was conducted with Crisprfinder [18].

**Table 1:** Top BLASTn hits for DSM 24701 16S rDNA gene sequence

	**Species**	**Accession**	**Score**	**Query coverage**	**E value**	**Max identity**
1	*Advenella kashmirensis* WT001	CP003555.1	2265	100%	0	93%
2	*Bordetella* sp. p23 (2011)	HQ652588.1	2255	99%	0	93%
3	Uncultured compost bacterium clone ASC718	JQ775330.1	2244	99%	0	93%
4	*Taylorella equigenitalis* 14/56	HE681423.1	2237	100%	0	93%
5	*Taylorella equigenitalis* ATCC 35865	CP003264.1	2237	100%	0	93%
6	*Taylorella equigenitalis* MCE9	CP002456.1	2237	100%	0	93%
7	*Bordetella* sp. d16	HQ652589.1	2235	98%	0	93%
8	*Achromobacter* sp. CH1	HQ619222.1	2231	99%	0	93%
9	*Achromobacter* sp. MT-E3	EU727196.1	2231	99%	0	93%

### Phylogenetic profile

An array was constructed containing rows of putative genes and columns of fully sequenced bacterial genomes, following the strategy of Wu and Eisen [19]. The absence and presence of a gene in the species is indicated by 0 or 1, as determined by BLASTp of the predicted genes from DSM 24701 against the SEED database of proteins from fully sequenced genomes with an E-value cut-off of 10E-05. Clusters were made using CLUSTER 3.0 with a complete linkage hierarchical analysis and weighting of the species in an attempt to remove phylogenetic bias, and visualized with JavaTreeview (both available at http://rana.lbl.gov/EisenSoftware.htm).

### Duplication analysis

BLASTp of the predicted protein sequences from DSM 24701 was performed against a database of the same set of sequences, to find duplicates inside the genome (paralogs). Reciprocal hits and self-hits were excluded, and BLAST results with an E-value cut-off of 10E-05, >150aa long, and >30% sequence identity were counted as duplicates, largely following the strategy of Gevers et al. [20]. We excluded all 57 sequences <150aa long in order to avoid overestimating the duplication rate by only including short sequences that do not have a paralog.

## Results and discussion

### Bacterium identification

At necropsy, the post mortem examination of the parakeet revealed that the liver had a marbled surface and the spleen was swollen. No other macroscopic lesions were observed. The histology revealed several abnormalities. The lungs had diffused alveolar edemas and congestion. The heart had multifocal epicardial and myocardial edemas. Spleen and liver had diffuse sinusoidal congestion and multifocal accumulation of histiocytes. Bacterial culture of the lung and liver revealed the presence of small Gram-negative, non-haemolytic, non-motile rods in the lung. Visible colonies of the bacterial strain (initially labeled JF4266 in the lab, and referred to as DSM 24701 in this paper) appeared after 2-day incubation at 37°C on blood agar plates in a 5% CO_2_-enriched atmosphere. The bacterium did not grow in LB broth or enriched Mycoplasma broth medium (Axcell Biotechnologies, St. Genis l’Argentière, France) at 37°C with and without 5% CO_2_. A detailed growth condition profile in comparison with *P. europaea, T. equigenitalis* and *T. asinigenitalis* is included in Additional file 1: Table S1. It shows that DSM 24701 and *P. europaea* grow in aerobic or capnophilic conditions at 30°C and 42°C. DSM 24701 interestingly does not grow at 37°C in aerobic conditions, but only in capnophilic conditions. The cytochrome oxidase and catalase spot tests were positive while indole was negative. Standard phenotypic analysis could not identify the isolate (Additional file 1: Table S1). The enzyme profile can differentiate DSM 24701 from the type strains of *P. europaea, T. equigenitalis* and *T. asinigenitalis* (Table 2). The major respiratory quinone of the strain DSM 24701 is Q8 and the major polar lipids are phosphatidylethanolamine, phosphatidylglycerol, two unknown phosphoaminolipids, two unknown phospholipids and two unknown aminolipids. The proportion of several cellular fatty acids from DSM 24701 is reported in Table 3.

**Table 2:** **Differential taxonomic characteristics between DSM 24701, *T*. *equigenitalis *** **(DSM 10668 T), *T*. *asinigenitalis *** **(CIP 79.7 T) and *P*. *europaea *** **(LMG 10982 T)**

**Enzyme**	***P*. *europaea *** **LMG 10982 T**	***T. asinigenitalis *** **CIP 79.7 T**	***T. equigenitalis *** **DSM 10668 T**	**DSM 24701**
**API ZYM results**^**a**^				
Alcaline phosphatase	1	5	5	-
Esterase	2	1	1	4
Esterase lipase	1	-	-	2
Lipase	2	-	-	-
Leucine arylamidase	5	5	5	5
Valine arylamidase	3	2	1	1
Cystin arylamidase	-	1	1	-
Acid phosphatase	2	3	4	1
Naphtol-AS-BI-phosphohydrolase	1	2	4	3
**API NH results**^**b**^				
Penicillinase	-	+	-	-
Ornithine decarboxylase	-	-	-	w
γ-glutamyl transferase	-	+	+	+

^**a**^API ZYM scores: -, no activity; 1, lowest activity; 5, highest activity.

The four strains gave no reaction for indol, trypsin, chymotrypsin, α-galactosidase, β-galactosidase, β-glucuronidase, α-glucosidase, β-glucosidase, N-acetyl-β-glucosaminidase, α-mannosidase and α-fucosidase, urease and prolin arylamidase.

^**b**^API NH results: -, negative; w, weakly positive; +, positive.

**Table 3:** Cellular fatty acid composition of DSM 24701

**Fatty acid composition**	**DSM 24701**
10:0	-
12:0	tr
14:0	6.92
14:1 w5c, 14:1 w5t or both	-
15:0	2.30
15:1 w8c	-
16:0	35.31
16:0(3-OH)	1.3
16:1 w5c	tr
17:1 w6c	1.23
18:0	1.09
18:1 w5c	tr
18:1 w7c	38
19:0 10-methyl	-
20:1 w9t	-
Summed feature 1	TR
Summed feature 2	9.47
Summed feature 3	1.07
Summed feature 5	tr

tr, trace amount (<1%); -, not detected.

Summed feature 1, 15:1 isoH, 15:1isoI, 13:0 3-OH, or any combination.

Summed feature 2, 12:0 ALDE, 14:0 3-OH, 16:1 iso I, or any combination.

Summed feature 3, 16:1 w7c and/or 15 iso 2-OH.

Summed feature 5, 18:2 w6,9c and/or 18:0 ANTE.

#### ***Description of Basilea gen. nov.***

*Basilea* (Ba.si.le’a L. fem. N. referring to the Swiss town Basel, where the type strain was isolated)

Cells are small, Gram-negative, non-motile rods. Oxidase-positive and grows in aerobic or capnophillic conditions. Visible colonies appear after 2 days growth on blood agar plates at 30-42°C with 5% CO_2_. The major respiratory quinone is Q8 and the major polar lipids are phosphatidylethanolamine, phosphatidylglycerol, two unknown phosphoaminolipids, two unknown phospholipids and two unknown aminolipids. The major fatty acids were C_16:0_ and C_18:1_*ω7c*; C_12:0_was only detected in trace amounts. The type species is *Basilea psittacipulmonis*. The DNA G + C content of the type strain of this type species is 40%.

#### ***Description of psittacipulmonis sp. nov.***

*B. psittacipulmonis* (psitt.a.ci.pul.mon’is named because the type and only known strain was isolated from the lung of a parakeet). The description is the same as for the genus, with the following additions. Grows at 30°C, 37°C and 42°C with 5% CO_2_, and in aerobic conditions at 30°C, and 42°C. Does not grow in LB broth or enriched Mycoplasma broth medium. Enzyme tests did not indicate a reaction forindol, trypsin, chymotrypsin, α-galactosidase, β-galactosidase, β-glucuronidase, α-glucosidase, β-glucosidase, N-acetyl-β-glucosaminidase, α-mannosidase and α-fucosidase, urease and prolin arylamidase, alkaline phosphatase, lipase, cystin arylamidase or penicillinase. However, the species exhibits strong enzyme activity of esterase, leucine arylamidase, naphtol-AS-BI-phosphohydrolase and γ-glutamyl transferase, and intermediate activity of esterase lipase, valine arylamidase, acid phosphatase and ornithine decarboxylase. The chemotaxonomic characteristics listed in the type strain genus apply to this strain.

The type strain is *B. psittacipulmonis* DSM 24701, isolated from the lungs of a parakeet from Basel, Switzerland (= CIP 110308 T, 16S rDNA gene sequence Genbank accession number JX412111 and GI 406042063).

### Distribution in the cages and homes of pet owners

We explored whether this microorganism is common in the environment of pet parakeets by conducting PCRs on environmental templates with PCR primers that are unique to the *B. psittacipulmonis*. Primers were designed to specifically amplify *the B. psittacipulmonis* 16S rDNA gene and several protein-coding genes that were considered unidentified on RAST, and did not yield any hits on BLAST in the nr/nt database. PCR amplification of sample templates from the drinking water and bottom of cages housing healthy parakeets from various pet stores and private homes using these primers were all negative, while positive samples obtained by artificial contamination of the same material with 1 ng of DSM 24701 genomic DNA were positive. This suggests that the DSM 24701 is not commonly found in the cages of healthy parakeets.

### Phylogenetic analysis

**Figure 1:**
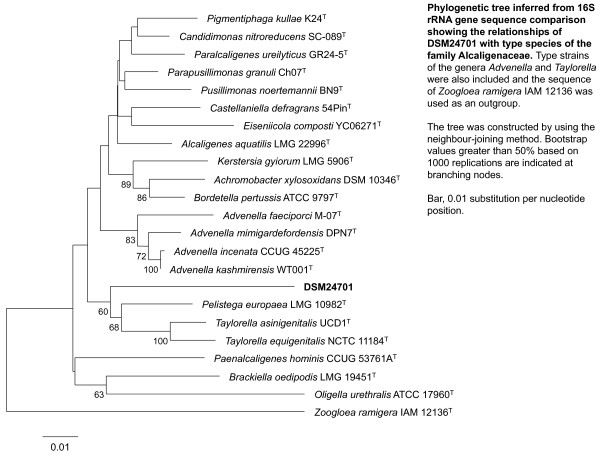
**Phylogenetic tree inferred from 16S rDNA gene sequence comparison showing the relationships of DSM 24701 with type species of the family *Alcaligenaceae*.** Type strains of species from the genera *Advenella* and *Taylorella* were also included and the sequence of *Zoogloea ramigera* IAM 12136 was used as an outgroup. The tree was constructed by using the neighbour-joining method. Bootstrap values greater than 50% based on 1000 replications are indicated at branching nodes. Bar, 0.01 substitution per nucleotide position.

Comparative phylogenetic analysis of 16S rDNA gene sequence with closely related species reveals that the bacterium is a *Betaproteobacterium* in the family *Alcaligenaceae*, closely related to members of the genus *Pelistega* and the genus *Taylorella* (Figure 1 contains neighbor joining tree, while Additional file 2: Figure S1 contains maximum likelihood and maximum parsimony trees). A neighbor joining tree of the *rpoB* gene sequence including *P. europaea* and several related taxa was also constructed (Additional file 3: Figure S2). The RDP naïve Bayesian Classifier assigns DSM 24701 to the family *Alcaligenaceae* with 100% confidence, but designates the strain as unclassified *Alcaligenaceae* with a 60% bootstrap confidence value for the genus *Pelistega*. The best match for the 16S rDNA gene sequence in the RDP and the NCBI has only 93% identity (Table 1). Because separation into bacterial genera typically occurs below 95% 16S rDNA gene sequence identity [21], the new isolate belongs to a new genus within the *Alcaligenaceae* family [22,23]. Similarly, the most closely related *rpoB* gene, from *P. europaea*, has only 76% identity (Additional file 1: Table S2). Separation into bacterial genera typically occurs below 85.5% *rpoB* gene identity [24]. The top 16S rDNA gene sequence BLAST hits from the all nucleotide nr/nt database are also from the *Alcaligenaceae* family (Table 1), although the top BLAST hits are not actually the closest phylogenetic neighbors [25] as determined with the phylogenetic trees shown in Figure 1 and Additional file 2: Figure S1 and Additional file 3: Figure S2. Phenotypic characteristics, GC content, 16S rDNA and *rpoB* gene identity all place the DSM 24701 close to *P. europaea* and *T. equigenitalis* (Table 4). The genome comparisons discussed below rely on members of the *Alcaligenaceae* family whose entire genomes have been sequenced, including two members of the genus *Taylorella* and several members of the genus *Bordetella* including *B. pertussis*, the organism that causes whooping cough.

**Table 4:** **Comparison of DSM 24701 with other betaproteobacteria including many members of the family *Alcaligenaceae***

**Strain**	**Shape**	**Gram**	**Genome size (Mb)**	**Coding sequences**	**GC %**	**Observed growth rate (hours)**
DSM 24701	Rod	Neg	1.9	1658^a^	40	n/a
*Pelistega europaea*	Pleomorphic	Neg	n/a	n/a	~43^d^	n/a
*Taylorella equigenitalis* MCE9	Coccobacillus	Neg	1.7^g^	1557	37^g^	n/a
*Taylorella asinigenitalis 14/45*	Coccobacillus	Neg	1.5^g^	1423	38^g^	n/a
*Bordetella avium*	Coccobacillus	Neg	3.7^b^	3417^b^3463^a^	62^b^	n/a
*Bordetella bronchiseptica*	Coccobacillus	Neg	5.3^b^	5011^b^5024^a^	68^b^	24-48^f^
*Bordetella pertussis*	Coccobacillus	Neg	4.1^b^	3816^b^3799^a^	68^b^	48-72^f^
*Bordetella parapertussis*	Coccobacillus	Neg	4.8^b^	4404^b^4452^a^	68^b^	48-72^f^
*Ralstonia solanacearum*	Rod	Neg	5.8^c^	5129^c^5172^a^	67^c^	n/a
*Acidovorax avenae* subsp*. citrulli*	Rod	Neg	5.4^e^	4709^e^4071^a^	69^e^	n/a
*Burkholderia ambifaria* AMMD	Rod	Neg	7.5^e^	6617^e^6275^a^	67^e^	n/a
*Burkholderia cenocepacia*	Rod	Neg	7^e^	6477^e^6142^a^	67^e^	n/a
*Advenella kashmirensis*	Coccoid	Neg	4.4^h^	4563^h^	54^h^	48-72^h^

^a^[15] → ^b^[8] → ^c^[26] → ^d^[27] → ^e^[2] → ^f^[28] → ^g^[9] → ^h^[29].

*P. europaea* has been found in the lungs, trachea, liver and spleen of acutely diseased pigeons; clinical observations have led microbiologists to conclude that it is a pathogenic organism [30]. Low GC content and small genome size, features which are shared by *P. europaea*, *Taylorella* spp*.,* and this novel bacterium DSM 24701 [31], are different from the closely related, fully sequenced members of the *Alcaligenaceae* family such as the *Bordetella* with higher GC content (62-68%) and genome size (3.7-5.3 Mb) (Table 4).

### Genomic analysis

We used high coverage sequence data (~350×) with short reads of 36 bases from Solexa-Illumina, generating 195 contigs, when assembled with Edena (Table 5). A 454 run with only 10× coverage yielded 977 contigs. Merging this assembly with the one that resulted from the Illumina paired-end data did not improve the contiguity. Moreover, some errors at homopolymers stretches [32] propagated into the merged assembly. Therefore we discarded this data for the rest of the analysis.

**Table 5:** Illumina sequencing data and assembly statistics of the draft genome

	
Number of reads	18596374
Read length	36
Average pairing distance (standard deviation)	117.8 (10.3)
Number of contigs	195
Average contig size	9.9 Kbp
N50	41.6 Kbp
Max contig size	99 Kbp
Total size*	1.93 Mb
Raw coverage	347×
RAST predicted coding sequences	1664
Contigs included in annotation	88
RNAs	45

*The genome size was also determined to be 2.2 Mb through Optical Mapping with the restriction enzyme *Nhe*I.

#### Genome size as determined by contig assembly and optical mapping is near 2 Mb

The size of the DSM 24701 genome is estimated to be near 2 Mb by both Solexa-Illumina and 454 sequencing in addition to the results of an optical map generated by electrophoresis of fragments generated by an *Nhe*I digest of the genomic DNA (results not presented). The large effort which would be required to complete the genome was not undertaken. The 195 contigs were submitted for Rapid Annotation using Subsystem Technology [15] (http://rast.nmpdr.org/). The annotation process found 1664 coding sequences on 88 contigs. The remaining contigs were shorter than the average gene length, suggesting that any gene which may occur on those contigs could be truncated and would be harder for gene-calling algorithms to identify. RAST describes each of the coding sequences as a protein expression gene (peg) numbered 1-1664 as they appear on the contigs which are ordered largest to smallest, i.e. peg.1 is the first gene on the largest contig.

#### Common protein coding marker genes and dinucleotide frequency recapitulate relationships found in 16S rDNA gene tree

**Figure 2:**
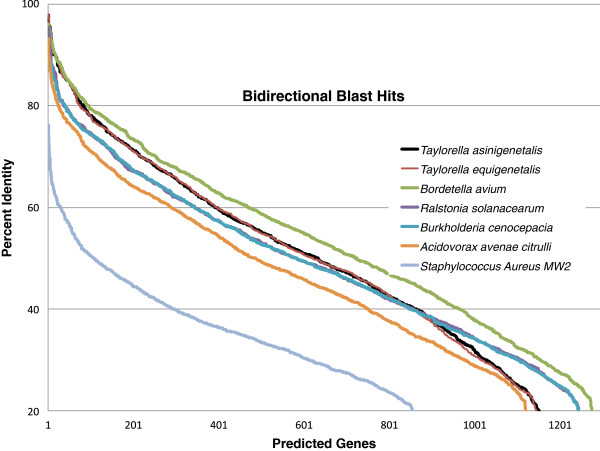
Bidirectional BLASTp hits between predicted genes for DSM 24701 and those of closely related fully sequenced genomes, calculated by RAST as the percent identity of the BLASTp hit (highest-scoring pair of segments).

The contigs were concatenated into a single molecule and analyzed with ML Tree (http://mltreemap.org/). This software searches through fully sequenced bacterial genomes for 31 common protein coding marker genes and constructs a phylogenetic tree based on the alignment of the best BLAST matches for these markers [33]. The draft genome of DSM 24701*,* containing all 31 marker genes on 10 different contigs, was the closest to the genomes of the *Bordetella* genus (data not shown) [34]. The best blast hits shown in Figure 2 also suggest that predicted genes from *Bordetella* have the highest sequence similarity with DSM 24701. Interestingly, dinucleotide usage analysis (shown in Additional file 4: Figure S3) recapitulates the phylogenetic relationships found with the 16S rDNA gene tree in Figure 1. Dinucleotide usage has a phylogenetic signature that has been shown to reflect the lifestyle and history of a micro-organism [35]. Five CRISPR sequences were identified using Crisprfinder, and two of them had significant blast scores (e value < 1e-27) with hypothetical proteins from the genus *Neisseria* and from *Pasteurella multocida* (Additional file 1: Table S4). Both *Neisseria* and *Pasteurella* can be part of the normal microbiota of humans and animals, while some species of these genera can cause infectious diseases.

#### Amino acid sequence homology shows that about a third of the predicted genes from DSM 24701 are shared with related genomes

**Figure 3:**
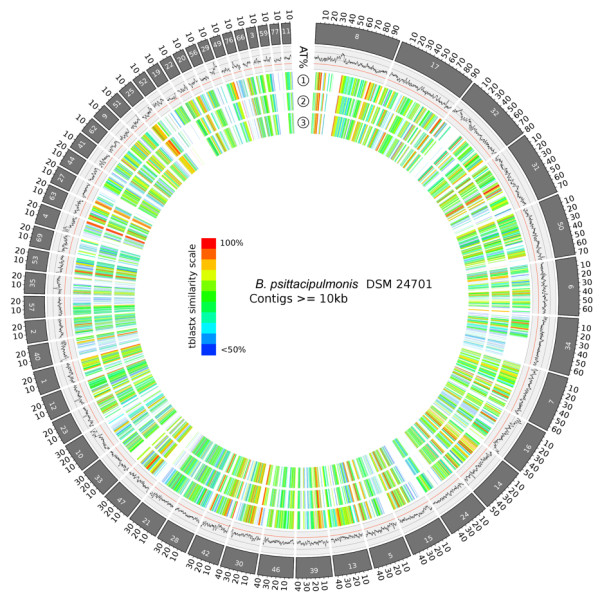
**Ring diagram showing blast similarity at the protein level and AT content.** External ring displays the DSM 24701 ordered contigs that are greater or equal to 10 kb. The AT% ring displays the AT% computed by using a sliding window of 1 kb. Axes range from 40% to 80%. Inner rings (1), (2) and (3) display the similarity scores at the protein level (tblastx, e-value cutoff 0.1). The compared species are (1) *B. avium* 197 N, (2) *T. asinigenitalis* MCE3 and (3) *T. equigenitalis* ATCC 35865.

Traditionally, bacterial species have been characterized since the 1960s using laborious DNA-DNA hybridization (DDH) with genomic DNA for related organisms, with a cut-off of 50-70% for members of the same species [36]. Now it is possible to compare the sequences of organisms with fully sequenced genomes, bypassing the need for DDH. Full genome sequence comparison methods such as Average Nucleotide Index (ANI) have been shown to be equivalent to DDH [36]. Species cutoff values of 70% DDH have been found to correspond to ANI values of 95% and 16S rDNA gene identity values of ~98% [36,37]. We attempted to make ANI calculations comparing the DSM 24701 sequence with the eight organisms with fully sequenced genomes listed in Figure 2, but found that the ANI calculations were only able to include about 20% of the genome sequence, and led to ANI values of approximately 65% [38]. There is not any fully sequenced genome that is similar enough to the DSM 24701 to allow for useful comparison by ANI or DDH. However, comparison of amino acid sequence homology of the predicted genes, as shown in Figure 2 by bidirectional BLAST hits taken from the RAST annotation [15], is a useful way to evaluate the similarities between the DSM 24701 and fully sequenced members of the *Alcaligenaceae* family. The top most similar genes (Additional file 1: Table S5) include highly conserved proteins, mostly ribosomal proteins. There are only a handful of proteins with >90% similarity when comparing this novel species with *B. avium, T. equigenitalis* and *T. asinigenitalis.* About a third of the putative genes from DSM 24701 have >50% identity with predicted genes from the genomes of *Bordetella* spp and *Taylorella* spp (Figure 2). The number of unique genes is quite large: 302 predicted genes have a BLAST identity <20% with the *B. avium, T. equigenitalis* and *T. asinigenitalis*. Most bacteria have a significant number of unique genes [39]; i.e. *T. asinigenitalis* has 141 genes absent from *T. equigenitalis*, and 359 genes not found in *B. avium*. The spectacular diversity of protein coding sequences in bacterial genomes is a major motivation for large-scale microbial sequencing efforts. Current tools allow us to map out potential functional characteristics of putative genes. However, it can be difficult to make meaningful conclusions about an organism that is not closely related to other sequenced organisms despite obtaining a nearly complete genome sequence. The ring diagram [40] in Figure 3 highlights the sparse homology with the closest sequenced genomes at the amino acid level.

#### GC Content analysis of concatenated DSM 24701 contigs suggests more recent genetic exchange with organisms that have low GC content

**Figure 4:**
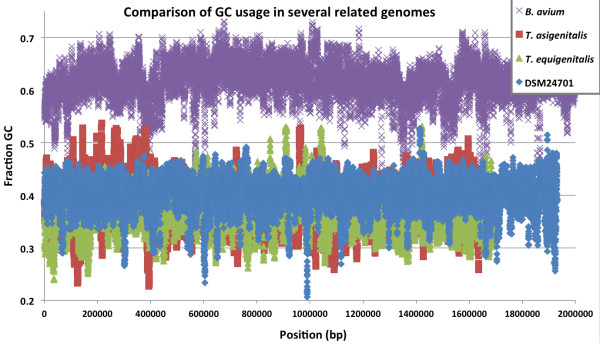
**Comparison of the GC content of the DSM 24701** ***, B. avium *** **197 N** ***, T. equigenitalis *** **and *T. asinigenitalis*.**

Comparison of the GC content of the DSM 24701 with that of *B. avium* 197 N*, T. equigenitalis* and *T. asinigenitalis* over the length of their respective genomes was conducted to look for variation which may indicate horizontal gene transfer (HGT). The DSM 24701 contigs were ordered from largest to smallest and fused into a single contiguous sequence, and the GC content of the four genomes shown in Figure 4 were analyzed in 100 bp windows with the Emboss isochore program [41]. The genome of DSM 24701 has consistently lower GC content than *B. avium* 197 N, and does not appear to have recent HGT events with organisms that have a GC content >60%, although there are several deviations of significant magnitude into regions of lower GC content. The *Taylorella* genomes and DSM 24701 have similar GC content. Shared GC content does not indicate greater overall homology; the *Taylorella* protein coding sequences do not share greater BLAST homology with DSM 24701 than *B. avium* (Figure 2).

#### Phylogenetic profiling yields a unique profile of gene clusters, some shared with Bordetella, phage or other respiratory pathogens

**Figure 5:**
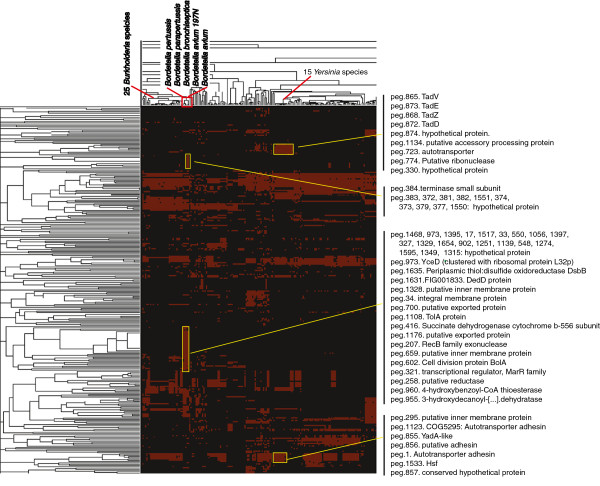
**Phylogenetic profile of the putative genes from DSM 24701** ***.***

We conducted a phylogenetic profile by constructing an array with rows consisting of the predicted genes of the DSM 24701 genome, and a column for each completely sequenced bacterium (Figure 5). A BLASTp query of the DSM 24701 predicted genes against a database containing all the genes from fully sequenced genomes in the SEED database was conducted to create a matrix with a 0 or 1 in each position depending on whether there was a BLASTp hit with a cutoff of 1E-5. The clusters of species recapitulate a phylogenetic tree (see Methods). The pattern of gene presence and absence for each species also leads to the formation of functionally related gene clusters. Visualization of this clustered array led to the observation of several interesting regions. For example, a cluster of at least eight putative genes including peg.872-4 involved in Type II/IV secretion are rarely present in any of the sequenced species, including *Bordetella*, but are consistently found in *Yersinia* species. A fraction of the genes are also found in other respiratory pathogens including *Haemophilus* and some potentially opportunistic *Shewanella* species (Figure 5). The GC content in this cluster is quite similar to that of the DSM 24701 genome, ranging from 36-40%. Another group of genes encoding bacterial adhesins and autotransporters (including peg.855 and peg.856, described as YadA-like, a well-studied *Yersinia* spp. protein known to play a role in host-pathogen interaction) is found in several respiratory pathogens, including many *Burkholderia* species, but does not have a single ortholog in the sequenced genomes of the *Bordetella* species. These examples illustrate that the DSM 24701 genome can be distinguished from the *Bordetella* species, and that it shares many genes thought to be important for respiratory pathogen species not belonging to the genus *Bordetella*.

There are also examples of gene clusters formed in the phylogenetic profile that are shared almost exclusively with the *Bordetella* species. Thirty-four genes in a cluster which is present consistently only in the *Bordetella* species are mostly described as hypothetical, but include genes predicted to be integral membrane proteins, TolA and a RecB-family exonuclease. Another intriguing cluster of 11 predicted genes that are all present in both genome sequences of *B. avium* encodes putative phage proteins, including the small terminase subunit involved in DNA packaging. Ten of the eleven genes in this cluster are located together on a contig of the DSM 24701 genome with the same gene order as the *Bordetella* species. We were surprised to find that the GC content of the DSM 24701 genes in this cluster ranged from 42-48%, while the orthologs from *Bordetella* species and several sequenced *Bordetella* phages have GC contents similar to that of their genomes just under 70%. The predicted phage terminase from DSM 24701 has 48% GC, which is high compared to the rest of the genome (Figure 4). It is interesting that this putative prophage cassette has such different GC content in DSM 24701 and *Bordetella* species; the difference may derive from a different phage or quick adaptation of the cassette sequence to a lower GC content in DSM 24701.

#### DSM 24701 shares some gene loss events with obligate intracellular bacteria

Of the 100 COGs lost by all obligate intracellular bacteria in a study of 317 genomes [12], only ~30 of them had equivalent representatives in the genome of strain DSM 24701 using the RAST annotation of predicted gene function. Strain DSM 24701 is not dependent on host cells; it is able to grow on blood agar plates. However, small genome size, high GC content and lack of ~70 genes also missing in obligate intracellular bacteria may indicate that DSM 24701 has taken steps on the one-way road toward gene loss like that which led other bacteria to become host dependent. Merkej et al [12] found that free living bacteria with larger genomes often have more genes that are described as virulence factors than pathogenic bacteria, challenging many early hypotheses that the presence of particular virulence factors was predictive of the pathogenicity of an organism [12]. In addition, HGT is more difficult for intracellular bacteria, which are isolated from encounters with genetically diverse microorganisms and phage. Mutations that affect gene regulation may also drive virulence in bacteria that can otherwise inhabit humans as harmless commensals, such *as Streptococcus pyogenes*[42,43], a bacterial species with similar genome size. Future annotation methods may become better at capturing these aspects of pathogenicity and bacterial lifestyle from genomic data.

#### Distribution of gene function annotation is similar to Taylorella genomes, and reflects the diverse repertoire of metabolic genes in DSM 24701

**Figure 6:**
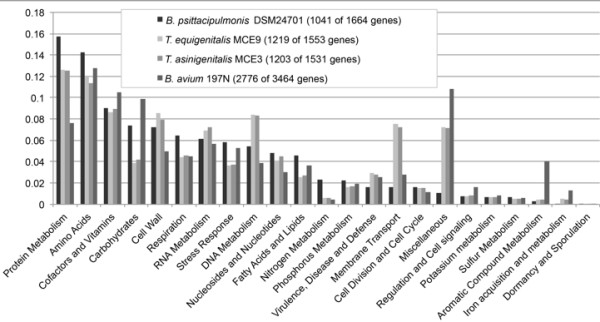
**Percentage of annotated genes assigned to functional categories by RAST for both DSM 24701 and *B. avium.***

Figure 6 shows the functional categories that RAST was able to assign to 1041 out of the 1664 predicted DSM 24701 genes, in comparison with the functional categories RAST assigned to *B. avium, T.equigenitalis* and *T. asinigenitalis*. The distribution for many categories is similar, especially for the closely related *Taylorella* genomes. There are some differences in the percentage of genes assigned to several metabolic categories – DSM 24701 is enriched for genes involved in protein, amino acid and nitrogen metabolism, along with carbohydrate and fatty acid metabolism and respiration, which suggest that DSM 24701 has maintained a diverse repertoire of metabolic genes. This may reflect the relative independence of DSM 24701 from the host, or a niche that requires broad metabolic capabilities.

#### DSM 24701 shares low duplication rate with bacteria of similar genome size

The presence of gene paralogs derived from duplication or HGT in bacteria is known to correspond to genome size and lifestyle. A comparison of duplication rates in 106 completely sequenced genomes in 2005 found that the paralogs represented, on average, 23.5 ± 8.7% of the predicted genes, ranging from 7% for *Rickettsia conorii* Malish 7 to 41% for *Streptomyces coelicolor* A3 (2) [20]. Using similar standards (see Methods) we found a duplication rate of 13% in DSM 24701. Low rates of duplication are associated with smaller genome size and host dependence. Table 6 shows the distribution of paralogs. Both peg.144 and peg.218 have seven paralogs in the genome, and they are both predicted to be ABC transporters, which are infamous for having large duplication rates.

**Table 6:** Distribution of gene paralogs within the DSM 24701genome

	
Singlets	1451
Pairs	135
Genes in 2 pairs	58
Genes in 3 pairs	13
Genes in 4 pairs	4
Genes in 7 pairs	2
Genes with paralogs (all pairs)	213 (13.3%)
Total genes (>150 bp)	1606
Excluded genes (<150 bp)	57

#### Shared gene homology varies widely inside bacterial families

Several recent genome comparison studies have drawn intriguing conclusions about genome evolution and organization. For example, the *Mycoplasma agalactiae* genome, long assumed to have undergone genome reduction in order to become one of the simplest free-living organisms with a minimal genome, was unexpectedly found to have a large fraction of predicted genes – 18% - likely acquired by HGT from species in distinct phylogenetic groups [31]. Sequencing of 16 *Mycoplasma* genomes allowed for detailed comparison between closely related species, revealing that the genomes are not very similar. For example, in a comparison of *M. agalactiae* strain PG2 with four other *Mycoplasma* genomes, no predicted genes with a blastp identity >90% were found, and only few (16%) with >50%. The genome of DSM 24701 is actually more similar to *Bordetella* species than this – about a third of the DSM 24701 genome has >50% identity with the sequenced *Taylorella* and *Bordetella* genomes (Figure 2).

In an attempt to better understand the biology of the newly discovered DSM 24701*,* and to intimate whether it is a pathogen, we also examined the putative genes that are unique to DSM 24701 in comparison to *B. avium, T. equigenitalis* and *T. asinigenitalis* (Additional file 1: Table S6). The unique genes include potential antibiotic resistance genes, CRISPR-related proteins, and members of the Tad (tight adherence gene) macromolecular transport system that may indicate that the secretion systems used by DSM 24701 are different (Additional file 5: Figure S4). This ancient secretion system is found in a long list of pathogenic genera, such as species belonging to the genera of *Haemophilus* and *Yersinia*. The *tad* genes found in many bacteria, including DSM 24701*,* are known to be involved in biofilm formation and colonization [44], which are essential in the first steps of infection by many bacterial pathogens.

## Conclusions

The organism described in our study (internal strain nr. JF4266, and referred to in this paper as DSM 24701) is different from the other genera belonging to the family *Alcaligenaceae*, according to phylogenetic, phenotypic and chemotaxonomic data. A new bacterial genus and species are proposed in order to place it taxonomically, with the name *Basilea psittacipulmonis* gen. nov., sp. nov. (originating from Basel, Switzerland and found in the lungs of *Psittacidae*). The presence of this easily cultured and yet unassigned bacterial strain, isolated from a common parakeet in a Basel petshop suggests that there may still be large parts of the bacterial kingdom which remain underexplored, even in the midst of the metagenomic revolution that has already yielded many *Proteobacteria* genome sequences.

The genomic sequence of a newly detected bacterium DSM 24701 will contribute to available sequence knowledge, with many genes that are not similar to any found in current databases. Sequence homology with related genomes, biochemical comparisons, dinucleotide usage, Crispr-detection and phylogenetic profiling allowed us to highlight several interesting features of this genome. However, as the passing of the 10 year anniversary of the human genome and our still vague understanding of its contents remind us, sequence information provides only limited biological knowledge of a live species. Additional sequence information from more closely related organisms would enable improved phylogenetic placement and, to some extent, functional characterization. Sequencing novel organisms – even an under-represented branch of a well-studied phyla - adds more unique information to the sequence databases, as recently shown by Jonathan Eisen and colleagues from the Genomic Encyclopedia of Bacteria and Archaea (GEBA) [3]. Although it is more difficult to analyze novel genomic sequence in comparative studies, the novel sequences may become starting material for unforeseen biotechnology projects or discoveries in microbial evolution.

### Data access

The assembled and annotated genome is publically on the RAST server with a guest account under the ID 666666.4954, and the 16S sequence has the Genbank accession number JX412111 and GI 406042063. 16S rDNA and *rpoB* gene alignments for phylogenetic tree construction can be found in the Dryad database: http://doi.org/10.5061/dryad.b341k.

## Competing interests

The authors declare that they have no competing interests.

## Authors’ contributions

KW analyzed the genome and wrote the manuscript, DH performed sequence analysis including assembly of sequence reads, LF performed the Illumina sequencing, JF and PP encountered the bacterium, performed phenotypic analyses and provided the original strain along with contributing ideas and discussion, NG constructed phylogenetic trees based on 16S rDNA gene sequences, VL conceived and performed the environmental sample PCRs on parakeet cage and water samples along with contributing extensive ideas and manuscript edits, and PF and JS supervised the project, contributing ideas and enthusiasm. All authors read and approved the final manuscript.

## Supplementary Material

Additional file 1: Table S1 Growth comparison of *Basilea psittacipulmonis* DSM 24701 and several closely related species in the conditions described. **Table S2.** BLASTn hits to the DSM24701 *rpoB* gene sequence for several related taxa also shown in the neighbor joining tree in Additional file 3: Figure S2. **Table S3.** Cellular fatty acid composition of DSM 24701. **Table S4.** Crisprs found in DSM24701. **Table S5.** Bidirectional BLASTp hits for all genes with greater than 90% identity between DSM 24701 and three of the closest fully sequenced genomes, *B. avium, Taylorella equigenitalis* and *T. asigenitalis.***Table S6.** A subset of the 302 predicted proteins from RAST annotation that are unique to DSM 24701 in a blast comparison of DSM 24701, *B. avium, T. equigenetalis and T. asigenitalis.* 179 of the 302 unique genes were annotated as hypothetical proteins, without a predicted function. Click here for file

Additional file 2: Figure S1 Phylogenetic trees based on maximum-likelihood (A) and maximum-parsimony (B) analyses of the rRNA gene sequences showing the relationships of DSM 24701 with type species of the family *Alcaligenaceae*. Type strains of the genera *Advenella* and *Taylorella* were also included and the sequence of *Zoogloea ramigera* IAM 12136 was used as an outgroup. Bootstrap values greater than 50% based on 1000 replications are indicated at branching nodes. Bar, 0.01 substitution per nucleotide position. Click here for file

Additional file 3: Figure S2 Phylogenetic tree inferred from *rpoB* gene sequence comparison showing the relationships of DSM24701 with selected members of the family *Alcaligenaceae*. All type species of the genera within the family *Alcaligenaceae*, for which the *rpoB* sequences were available, were included. For the genera *Advenella* and *Pusillimonas*, non-type species were included. The genus *Taylorella* was represented by the type species (*T. equigenitalis*) and a non-type species (*T. asinigenitalis*). Whenever possible, the type strains of the species were used. The sequence of *Dechloromonas aromatica* RCB was used as an outgroup. The tree was constructed by using the neighbour-joining method. Bootstrap values greater than 50% based on 1000 replications are indicated at branching nodes. Bar, 0.05 s. Click here for file

Additional file 4: Figure S3 Dinucleotide usage profile of DSM 24701 and several closely related fully sequenced bacteria. Multi-Dimensional scaling of a Bray-Curtis distance matrix of dinucleotide abundance tables is shown. Click here for file

Additional file 5: Figure S4 The *tad* locus on contig 35 of the DSM 24701*.* Numbered genes code for: 1. TadA, 2. RcpA, 3. TadB, 4. TadC, 5. TadZ, 6. TadD, 7. TadV, 8. Hypothetical protein, 9. Putative membrane protein, 10. RcpC, 11 Putative membrane protein, 12. TolR. Click here for file
