# Supplementary material for: A genomic perspective on a new bacterial genus and species from the Alcaligenaceae family, Basilea psittacipulmonis
Source: BMC Genomics. 2014 Mar 1;15:169. doi: 10.1186/1471-2164-15-169 (PMC4028982; doi:10.1186/1471-2164-15-169)
Supplement: Additional file 1: Table S1 — Growth comparison of Basilea psittacipulmonis DSM 24701 and several closely related species in the conditions described. Table S2. BLASTn hits to the DSM24701 rpoB gene sequence for several related taxa also shown in the neighbor joining tree in Additional file 3: Figure S2. Table S3. Cellular fatty acid composition of DSM 24701. Table S4. Crisprs found in DSM24701. Table S5. Bidirectional BLASTp hits for all genes with greater than 90% identity between DSM 24701 and three of the closest fully sequenced genomes, B. avium, Taylorella equigenitalis and T. asigenitalis.Table S6. A subset of the 302 predicted proteins from RAST annotation that are unique to DSM 24701 in a blast comparison of DSM 24701, B. avium, T. equigenetalis and T. asigenitalis. 179 of the 302 unique genes were annotated as hypothetical proteins, without a predicted function. [file 1471-2164-15-169-S1.DOC]

**Additional file 1: Table S1. Growth comparison of** *Basilea psittacipulmonis* DSM 24701, *T*. *equigenitalis* (DSM 10668 T), *T*. *asinigenitalis* (CIP 79.7 T) and *P*. *europaea* (LMG 10982 T).

| **ID-number** | **12-531** | **12-532** | **12-533** | **12-534** |
| --- | --- | --- | --- | --- |
|  | *Pelistega europaea LMG* 10982 T | *Taylorella asinigenitalis* CIP 107673 T | *Taylorella equigenitalis DSM* 10668 T | *Basilea psittacipulmonis* DSM 24701 |
|  |  |  |  |  |
| Aminopeptidase | + | + | + | + |
| Lyse with 3% KOH | + | + | + | + |
| Katalase | + | + | + | + |
| Oxidase | + | + | + | + |
|  |  |  |  |  |
| 24°C aerobic | - | - | - | - |
| 24°C anaerobic | - | w | w | - |
| 24°C microaerophilic | - | + | + | - |
| 24°C capnophilic | - | + | w | - |
|  |  |  |  |  |
| 30°C aerobic | + | - | w | + |
| 30°C anaerobic | - | ? | - | - |
| 30°C microaerophilic | - | + | + | - |
| 30°C capnophilic | + | ++ | ++ | + |
|  |  |  |  |  |
| 37°C aerobic | + | + | w | - |
| 37°C anaerobic | - | + | + | ? |
| 37°C microaerophilic | - | ? | w | - |
| 37°C capnophilic | + | + | + | + |
|  |  |  |  |  |
| 42°C aerobic | + | - | - | + |
| 42°C anaerobic | - | - | - | ? |
| 42°C microaerophilic | - | - | - | ? |
| 42°C capnophilic | + | + | - | + |
|  |  |  |  |  |

++

+

w

?

-

**Additional file 1: Table S2.** BLASTn hits to the DSM24701 rpoB gene sequence for several related taxa also shown in the neighbor joining tree in Supplemental Figure 2

|  | [**Max score**](http://blast.ncbi.nlm.nih.gov/Blast.cgi?CMD=Get&ALIGNDB_BATCH_ID=215268771&ALIGNDB_CGI_HOST=blast.be-md.ncbi.nlm.nih.gov&ALIGNDB_CGI_PATH=/ALIGNDB/alndb_asn.cgi&ALIGNDB_MASTER_ALIAS=SD_ALIGNDB_MASTER&ALIGNDB_MAX_ROWS=250&ALIGNDB_ORDER_CLAUSE=seq_evalue asc,aln_id asc&ALIGNDB_WHERE_CLAUSE=seq_evalue is not null&ALIGNMENTS=100&ALIGNMENT_VIEW=Pairwise&DATABASE_SORT=0&DESCRIPTIONS=250&DYNAMIC_FORMAT=on&FIRST_QUERY_NUM=0&FORMAT_OBJECT=Alignment&FORMAT_PAGE_TARGET=&FORMAT_TYPE=HTML&GET_SEQUENCE=yes&I_THRESH=&LINE_LENGTH=60&MASK_CHAR=2&MASK_COLOR=1&NUM_OVERVIEW=100&OLD_BLAST=false&PAGE=Nucleotides&QUERY_INDEX=0&QUERY_NUMBER=0&RESULTS_PAGE_TARGET=&RID=AH94DFZB014&SHOW_LINKOUT=yes&SHOW_OVERVIEW=yes&STEP_NUMBER=&USE_ALIGNDB=true&WORD_SIZE=11&OLD_VIEW=false&DISPLAY_SORT=1&HSP_SORT=1) | [**Total score**](http://blast.ncbi.nlm.nih.gov/Blast.cgi?CMD=Get&ALIGNDB_BATCH_ID=215268771&ALIGNDB_CGI_HOST=blast.be-md.ncbi.nlm.nih.gov&ALIGNDB_CGI_PATH=/ALIGNDB/alndb_asn.cgi&ALIGNDB_MASTER_ALIAS=SD_ALIGNDB_MASTER&ALIGNDB_MAX_ROWS=250&ALIGNDB_ORDER_CLAUSE=seq_evalue asc,aln_id asc&ALIGNDB_WHERE_CLAUSE=seq_evalue is not null&ALIGNMENTS=100&ALIGNMENT_VIEW=Pairwise&DATABASE_SORT=0&DESCRIPTIONS=250&DYNAMIC_FORMAT=on&FIRST_QUERY_NUM=0&FORMAT_OBJECT=Alignment&FORMAT_PAGE_TARGET=&FORMAT_TYPE=HTML&GET_SEQUENCE=yes&I_THRESH=&LINE_LENGTH=60&MASK_CHAR=2&MASK_COLOR=1&NUM_OVERVIEW=100&OLD_BLAST=false&PAGE=Nucleotides&QUERY_INDEX=0&QUERY_NUMBER=0&RESULTS_PAGE_TARGET=&RID=AH94DFZB014&SHOW_LINKOUT=yes&SHOW_OVERVIEW=yes&STEP_NUMBER=&USE_ALIGNDB=true&WORD_SIZE=11&OLD_VIEW=false&DISPLAY_SORT=2&HSP_SORT=1) | [**Query cover**](http://blast.ncbi.nlm.nih.gov/Blast.cgi?CMD=Get&ALIGNDB_BATCH_ID=215268771&ALIGNDB_CGI_HOST=blast.be-md.ncbi.nlm.nih.gov&ALIGNDB_CGI_PATH=/ALIGNDB/alndb_asn.cgi&ALIGNDB_MASTER_ALIAS=SD_ALIGNDB_MASTER&ALIGNDB_MAX_ROWS=250&ALIGNDB_ORDER_CLAUSE=seq_evalue asc,aln_id asc&ALIGNDB_WHERE_CLAUSE=seq_evalue is not null&ALIGNMENTS=100&ALIGNMENT_VIEW=Pairwise&DATABASE_SORT=0&DESCRIPTIONS=250&DYNAMIC_FORMAT=on&FIRST_QUERY_NUM=0&FORMAT_OBJECT=Alignment&FORMAT_PAGE_TARGET=&FORMAT_TYPE=HTML&GET_SEQUENCE=yes&I_THRESH=&LINE_LENGTH=60&MASK_CHAR=2&MASK_COLOR=1&NUM_OVERVIEW=100&OLD_BLAST=false&PAGE=Nucleotides&QUERY_INDEX=0&QUERY_NUMBER=0&RESULTS_PAGE_TARGET=&RID=AH94DFZB014&SHOW_LINKOUT=yes&SHOW_OVERVIEW=yes&STEP_NUMBER=&USE_ALIGNDB=true&WORD_SIZE=11&OLD_VIEW=false&DISPLAY_SORT=4&HSP_SORT=0) | [**E value**](http://blast.ncbi.nlm.nih.gov/Blast.cgi?CMD=Get&ALIGNDB_BATCH_ID=215268771&ALIGNDB_CGI_HOST=blast.be-md.ncbi.nlm.nih.gov&ALIGNDB_CGI_PATH=/ALIGNDB/alndb_asn.cgi&ALIGNDB_MASTER_ALIAS=SD_ALIGNDB_MASTER&ALIGNDB_MAX_ROWS=250&ALIGNDB_ORDER_CLAUSE=seq_evalue asc,aln_id asc&ALIGNDB_WHERE_CLAUSE=seq_evalue is not null&ALIGNMENTS=100&ALIGNMENT_VIEW=Pairwise&DATABASE_SORT=0&DESCRIPTIONS=250&DYNAMIC_FORMAT=on&FIRST_QUERY_NUM=0&FORMAT_OBJECT=Alignment&FORMAT_PAGE_TARGET=&FORMAT_TYPE=HTML&GET_SEQUENCE=yes&I_THRESH=&LINE_LENGTH=60&MASK_CHAR=2&MASK_COLOR=1&NUM_OVERVIEW=100&OLD_BLAST=false&PAGE=Nucleotides&QUERY_INDEX=0&QUERY_NUMBER=0&RESULTS_PAGE_TARGET=&RID=AH94DFZB014&SHOW_LINKOUT=yes&SHOW_OVERVIEW=yes&STEP_NUMBER=&USE_ALIGNDB=true&WORD_SIZE=11&OLD_VIEW=false&DISPLAY_SORT=0&HSP_SORT=0) | [**Ident**](http://blast.ncbi.nlm.nih.gov/Blast.cgi?CMD=Get&ALIGNDB_BATCH_ID=215268771&ALIGNDB_CGI_HOST=blast.be-md.ncbi.nlm.nih.gov&ALIGNDB_CGI_PATH=/ALIGNDB/alndb_asn.cgi&ALIGNDB_MASTER_ALIAS=SD_ALIGNDB_MASTER&ALIGNDB_MAX_ROWS=250&ALIGNDB_ORDER_CLAUSE=seq_evalue asc,aln_id asc&ALIGNDB_WHERE_CLAUSE=seq_evalue is not null&ALIGNMENTS=100&ALIGNMENT_VIEW=Pairwise&DATABASE_SORT=0&DESCRIPTIONS=250&DYNAMIC_FORMAT=on&FIRST_QUERY_NUM=0&FORMAT_OBJECT=Alignment&FORMAT_PAGE_TARGET=&FORMAT_TYPE=HTML&GET_SEQUENCE=yes&I_THRESH=&LINE_LENGTH=60&MASK_CHAR=2&MASK_COLOR=1&NUM_OVERVIEW=100&OLD_BLAST=false&PAGE=Nucleotides&QUERY_INDEX=0&QUERY_NUMBER=0&RESULTS_PAGE_TARGET=&RID=AH94DFZB014&SHOW_LINKOUT=yes&SHOW_OVERVIEW=yes&STEP_NUMBER=&USE_ALIGNDB=true&WORD_SIZE=11&DISPLAY_SORT=3&HSP_SORT=3) | **Accession** |
| --- | --- | --- | --- | --- | --- | --- |
| ***[Pelistega europaea](http://blast.ncbi.nlm.nih.gov/Blast.cgi#alnHdr_289468048)* strain IPDH212/90 RpoB (rpoB) gene, partial cds** | 302 | 302 | 10% | 2.00E-84 | 76% | [FJ999741.1](http://www.ncbi.nlm.nih.gov/nucleotide/289468048?report=genbank&log$=nucltop&blast_rank=1&RID=B2U7BDHX01R) |
| ***Advenella kashmirensis* WT001, complete genome** | 2120 | 2120 | 100% | 0 | 72% | CP003555.1 |
| ***[Taylorella asinigenitalis](http://blast.ncbi.nlm.nih.gov/Blast.cgi#alnHdr_347973256)* MCE3, complete genome** | 2046 | 2046 | 99% | 0 | 72% | [CP003059.1](http://www.ncbi.nlm.nih.gov/nucleotide/347973256?report=genbank&log$=nucltop&blast_rank=2&RID=AH94DFZB014) |
| ***[Taylorella asinigenitalis](http://blast.ncbi.nlm.nih.gov/Blast.cgi#alnHdr_399116003)* 14/45 draft genome** | 2044 | 2044 | 99% | 0 | 71% | [HE681424.1](http://www.ncbi.nlm.nih.gov/nucleotide/399116003?report=genbank&log$=nucltop&blast_rank=3&RID=AH94DFZB014) |
| ***[Taylorella equigenitalis](http://blast.ncbi.nlm.nih.gov/Blast.cgi#alnHdr_394349192)* ATCC 35865, complete genome** | 1867 | 1867 | 99% | 0 | 71% | [CP003264.1](http://www.ncbi.nlm.nih.gov/nucleotide/394349192?report=genbank&log$=nucltop&blast_rank=4&RID=AH94DFZB014) |
| ***[Taylorella equigenitalis](http://blast.ncbi.nlm.nih.gov/Blast.cgi#alnHdr_399114527)* 14/56 draft genome** | 1862 | 1862 | 99% | 0 | 71% | [HE681423.1](http://www.ncbi.nlm.nih.gov/nucleotide/399114527?report=genbank&log$=nucltop&blast_rank=5&RID=AH94DFZB014) |
| ***[Taylorella equigenitalis](http://blast.ncbi.nlm.nih.gov/Blast.cgi#alnHdr_317108213)* MCE9, complete genome** | 1862 | 1862 | 99% | 0 | 71% | [CP002456.1](http://www.ncbi.nlm.nih.gov/nucleotide/317108213?report=genbank&log$=nucltop&blast_rank=6&RID=AH94DFZB014) |
| ***[Bordetella avium](http://blast.ncbi.nlm.nih.gov/Blast.cgi#alnHdr_115421100)* 197N complete genome** | 1613 | 1613 | 96% | 0 | 69% | [AM167904.1](http://www.ncbi.nlm.nih.gov/nucleotide/115421100?report=genbank&log$=nucltop&blast_rank=10&RID=AH94DFZB014) |
| ***[Bordetella parapertussis](http://blast.ncbi.nlm.nih.gov/Blast.cgi#alnHdr_408440925)* Bpp5 complete genome** | 1202 | 1492 | 87% | 0 | 69% | [HE965803.1](http://www.ncbi.nlm.nih.gov/nucleotide/408440925?report=genbank&log$=nucltop&blast_rank=61&RID=AH94DFZB014) |
| ***[Ralstonia solanacearum](http://blast.ncbi.nlm.nih.gov/Blast.cgi#alnHdr_334194119)* Po82, complete genome** | 1032 | 1221 | 92% | 0 | 67% | [CP002819.1](http://www.ncbi.nlm.nih.gov/nucleotide/334194119?report=genbank&log$=nucltop&blast_rank=92&RID=AH94DFZB014) |

**Additional file 1: Table S3.** Cellular fatty acid composition of DSM 24701

| **Fatty acid composition** | **DSM 24701** |
| --- | --- |
| 10:0 | - |
| 12:0 | tr |
| 14:0 | 6.92 |
| 14:1 w5c, 14:1 w5t or both | - |
| 15:0 | 2.30 |
| 15:1 w8c | - |
| 16:0 | 35.31 |
| 16:0(3-OH) | 1.3 |
| 16:1 w5c | tr |
| 17:1 w6c | 1.23 |
| 18:0 | 1.09 |
| 18:1 w5c | tr |
| 18:1 w7c | 38 |
| 19:0 10-methyl | - |
| 20:1 w9t | - |
| Summed feature 1 | TR |
| Summed feature 2 | 9.47 |
| Summed feature 3 | 1.07 |
| Summed feature 5 | tr |

tr, trace amount (<1%); -, not detected.

Summed feature 1, 15:1 isoH, 15:1isoI, 13:0 3-OH, or any combination.

Summed feature 2, 12:0 ALDE, 14:0 3-OH, 16:1 iso I, or any combination.

Summed feature 3, 16:1 w7c and/or 15 iso 2-OH.

Summed feature 5, 18:2 w6,9c and/or 18:0 ANTE.

**Additional file 1: Table S4**: Crisprs found in DSM24701

| **CRISPR id : tmp_1_Crispr_2** |
| --- |
| - CRISPR start position : 415980 ---------- CRISPR end position : 416674 ---------- CRISPR length : 694 |
| - DR consensus : GTTGTAGTTTCCTCTCTCATCTCGTAATGCTACAAT |
| - DR length : 36 Number of spacers : 10 |
| | **Accession** | **Description** | **[Max score](http://blast.ncbi.nlm.nih.gov/Blast.cgi?CMD=Get&ALIGNMENTS=100&ALIGNMENT_VIEW=Pairwise&DATABASE_SORT=0&DESCRIPTIONS=100&FIRST_QUERY_NUM=0&FORMAT_OBJECT=Alignment&FORMAT_PAGE_TARGET=&FORMAT_TYPE=HTML&GET_SEQUENCE=yes&I_THRESH=&MASK_CHAR=2&MASK_COLOR=1&NEW_VIEW=yes&NUM_OVERVIEW=100&NUM_QUERIES=5&OLD_BLAST=false&PAGE=Nucleotides&QUERY_INDEX=4&QUERY_NUMBER=0&RESULTS_PAGE_TARGET=&RID=AYNU7BGD016&SHOW_LINKOUT=yes&SHOW_OVERVIEW=yes&STEP_NUMBER=&WORD_SIZE=11&DISPLAY_SORT=1&HSP_SORT=1#sort_mark)** | **[Total score](http://blast.ncbi.nlm.nih.gov/Blast.cgi?CMD=Get&ALIGNMENTS=100&ALIGNMENT_VIEW=Pairwise&DATABASE_SORT=0&DESCRIPTIONS=100&FIRST_QUERY_NUM=0&FORMAT_OBJECT=Alignment&FORMAT_PAGE_TARGET=&FORMAT_TYPE=HTML&GET_SEQUENCE=yes&I_THRESH=&MASK_CHAR=2&MASK_COLOR=1&NEW_VIEW=yes&NUM_OVERVIEW=100&NUM_QUERIES=5&OLD_BLAST=false&PAGE=Nucleotides&QUERY_INDEX=4&QUERY_NUMBER=0&RESULTS_PAGE_TARGET=&RID=AYNU7BGD016&SHOW_LINKOUT=yes&SHOW_OVERVIEW=yes&STEP_NUMBER=&WORD_SIZE=11&DISPLAY_SORT=2&HSP_SORT=1#sort_mark)** | **[Query coverage](http://blast.ncbi.nlm.nih.gov/Blast.cgi?CMD=Get&ALIGNMENTS=100&ALIGNMENT_VIEW=Pairwise&DATABASE_SORT=0&DESCRIPTIONS=100&FIRST_QUERY_NUM=0&FORMAT_OBJECT=Alignment&FORMAT_PAGE_TARGET=&FORMAT_TYPE=HTML&GET_SEQUENCE=yes&I_THRESH=&MASK_CHAR=2&MASK_COLOR=1&NEW_VIEW=yes&NUM_OVERVIEW=100&NUM_QUERIES=5&OLD_BLAST=false&PAGE=Nucleotides&QUERY_INDEX=4&QUERY_NUMBER=0&RESULTS_PAGE_TARGET=&RID=AYNU7BGD016&SHOW_LINKOUT=yes&SHOW_OVERVIEW=yes&STEP_NUMBER=&WORD_SIZE=11&DISPLAY_SORT=4&HSP_SORT=0#sort_mark)** | **[E value](http://blast.ncbi.nlm.nih.gov/Blast.cgi?CMD=Get&ALIGNMENTS=100&ALIGNMENT_VIEW=Pairwise&DATABASE_SORT=0&DESCRIPTIONS=100&FIRST_QUERY_NUM=0&FORMAT_OBJECT=Alignment&FORMAT_PAGE_TARGET=&FORMAT_TYPE=HTML&GET_SEQUENCE=yes&I_THRESH=&MASK_CHAR=2&MASK_COLOR=1&NEW_VIEW=yes&NUM_OVERVIEW=100&NUM_QUERIES=5&OLD_BLAST=false&PAGE=Nucleotides&QUERY_INDEX=4&QUERY_NUMBER=0&RESULTS_PAGE_TARGET=&RID=AYNU7BGD016&SHOW_LINKOUT=yes&SHOW_OVERVIEW=yes&STEP_NUMBER=&WORD_SIZE=11&DISPLAY_SORT=0&HSP_SORT=0#sort_mark)** | **[Max ident](http://blast.ncbi.nlm.nih.gov/Blast.cgi?CMD=Get&ALIGNMENTS=100&ALIGNMENT_VIEW=Pairwise&DATABASE_SORT=0&DESCRIPTIONS=100&FIRST_QUERY_NUM=0&FORMAT_OBJECT=Alignment&FORMAT_PAGE_TARGET=&FORMAT_TYPE=HTML&GET_SEQUENCE=yes&I_THRESH=&MASK_CHAR=2&MASK_COLOR=1&NEW_VIEW=yes&NUM_OVERVIEW=100&NUM_QUERIES=5&OLD_BLAST=false&PAGE=Nucleotides&QUERY_INDEX=4&QUERY_NUMBER=0&RESULTS_PAGE_TARGET=&RID=AYNU7BGD016&SHOW_LINKOUT=yes&SHOW_OVERVIEW=yes&STEP_NUMBER=&WORD_SIZE=11&DISPLAY_SORT=3&HSP_SORT=3#sort_mark)** |  | | --- | --- | --- | --- | --- | --- | --- | --- | | [FR774048.1](http://www.ncbi.nlm.nih.gov/nucleotide/319409534?report=genbank&log$=nucltop&blast_rank=1&RID=AYNU7BGD016) | Neisseria meningitidis WUE 2594 complete genome | [131](http://blast.ncbi.nlm.nih.gov/Blast.cgi#319409534) | 1321 | 98% | 4e-27 | 85% |  | |
|  |
| | Top of Form  Bottom of Form | Top of Form  Bottom of Form | Top of Form  Bottom of Form | Top of Form  Bottom of Form | | --- | --- | --- | --- | |
| **CRISPR id : tmp_1_Crispr_11** |
| - CRISPR start position : 1483157 ---------- CRISPR end position : 1483456 ---------- CRISPR length : 299 |
| - DR consensus : GTATCCGAATCCCTTTGCAATCAGGGAGTCTATTCT |
| - DR length : 36 Number of spacers : 4 |
|  |
| | Top of Form  Bottom of Form | Top of Form  Bottom of Form | Top of Form  Bottom of Form | Top of Form  Bottom of Form | | --- | --- | --- | --- | |
| **CRISPR id : tmp_1_Crispr_17** |
| - CRISPR start position : 1882013 ---------- CRISPR end position : 1882839 ---------- CRISPR length : 826 |
| - DR consensus : AGAATAGACTCCCTGATTGCAAAGGGATTCGGATAC |
| - DR length : 36 Number of spacers : 12 |
|  |
| | Top of Form  Bottom of Form | Top of Form  Bottom of Form | Top of Form  Bottom of Form | Top of Form  Bottom of Form | | --- | --- | --- | --- | |
| **CRISPR id : tmp_1_Crispr_18** |
| - CRISPR start position : 1890069 ---------- CRISPR end position : 1890500 ---------- CRISPR length : 431 |
| - DR consensus : GTTGTAGTTTCCTCTCTCATCTCGTAATGCTACAAT |
| - DR length : 36 Number of spacers : 6 |
| | **Accession** | **Description** | **[Max score](http://blast.ncbi.nlm.nih.gov/Blast.cgi?CMD=Get&ALIGNMENTS=100&ALIGNMENT_VIEW=Pairwise&DATABASE_SORT=0&DESCRIPTIONS=100&FIRST_QUERY_NUM=0&FORMAT_OBJECT=Alignment&FORMAT_PAGE_TARGET=&FORMAT_TYPE=HTML&GET_SEQUENCE=yes&I_THRESH=&MASK_CHAR=2&MASK_COLOR=1&NEW_VIEW=yes&NUM_OVERVIEW=100&NUM_QUERIES=5&OLD_BLAST=false&PAGE=Nucleotides&QUERY_INDEX=2&QUERY_NUMBER=0&RESULTS_PAGE_TARGET=&RID=AYNU7BGD016&SHOW_LINKOUT=yes&SHOW_OVERVIEW=yes&STEP_NUMBER=&WORD_SIZE=11&DISPLAY_SORT=1&HSP_SORT=1#sort_mark)** | **[Total score](http://blast.ncbi.nlm.nih.gov/Blast.cgi?CMD=Get&ALIGNMENTS=100&ALIGNMENT_VIEW=Pairwise&DATABASE_SORT=0&DESCRIPTIONS=100&FIRST_QUERY_NUM=0&FORMAT_OBJECT=Alignment&FORMAT_PAGE_TARGET=&FORMAT_TYPE=HTML&GET_SEQUENCE=yes&I_THRESH=&MASK_CHAR=2&MASK_COLOR=1&NEW_VIEW=yes&NUM_OVERVIEW=100&NUM_QUERIES=5&OLD_BLAST=false&PAGE=Nucleotides&QUERY_INDEX=2&QUERY_NUMBER=0&RESULTS_PAGE_TARGET=&RID=AYNU7BGD016&SHOW_LINKOUT=yes&SHOW_OVERVIEW=yes&STEP_NUMBER=&WORD_SIZE=11&DISPLAY_SORT=2&HSP_SORT=1#sort_mark)** | **[Query coverage](http://blast.ncbi.nlm.nih.gov/Blast.cgi?CMD=Get&ALIGNMENTS=100&ALIGNMENT_VIEW=Pairwise&DATABASE_SORT=0&DESCRIPTIONS=100&FIRST_QUERY_NUM=0&FORMAT_OBJECT=Alignment&FORMAT_PAGE_TARGET=&FORMAT_TYPE=HTML&GET_SEQUENCE=yes&I_THRESH=&MASK_CHAR=2&MASK_COLOR=1&NEW_VIEW=yes&NUM_OVERVIEW=100&NUM_QUERIES=5&OLD_BLAST=false&PAGE=Nucleotides&QUERY_INDEX=2&QUERY_NUMBER=0&RESULTS_PAGE_TARGET=&RID=AYNU7BGD016&SHOW_LINKOUT=yes&SHOW_OVERVIEW=yes&STEP_NUMBER=&WORD_SIZE=11&DISPLAY_SORT=4&HSP_SORT=0#sort_mark)** | **[E value](http://blast.ncbi.nlm.nih.gov/Blast.cgi?CMD=Get&ALIGNMENTS=100&ALIGNMENT_VIEW=Pairwise&DATABASE_SORT=0&DESCRIPTIONS=100&FIRST_QUERY_NUM=0&FORMAT_OBJECT=Alignment&FORMAT_PAGE_TARGET=&FORMAT_TYPE=HTML&GET_SEQUENCE=yes&I_THRESH=&MASK_CHAR=2&MASK_COLOR=1&NEW_VIEW=yes&NUM_OVERVIEW=100&NUM_QUERIES=5&OLD_BLAST=false&PAGE=Nucleotides&QUERY_INDEX=2&QUERY_NUMBER=0&RESULTS_PAGE_TARGET=&RID=AYNU7BGD016&SHOW_LINKOUT=yes&SHOW_OVERVIEW=yes&STEP_NUMBER=&WORD_SIZE=11&DISPLAY_SORT=0&HSP_SORT=0#sort_mark)** | **[Max ident](http://blast.ncbi.nlm.nih.gov/Blast.cgi?CMD=Get&ALIGNMENTS=100&ALIGNMENT_VIEW=Pairwise&DATABASE_SORT=0&DESCRIPTIONS=100&FIRST_QUERY_NUM=0&FORMAT_OBJECT=Alignment&FORMAT_PAGE_TARGET=&FORMAT_TYPE=HTML&GET_SEQUENCE=yes&I_THRESH=&MASK_CHAR=2&MASK_COLOR=1&NEW_VIEW=yes&NUM_OVERVIEW=100&NUM_QUERIES=5&OLD_BLAST=false&PAGE=Nucleotides&QUERY_INDEX=2&QUERY_NUMBER=0&RESULTS_PAGE_TARGET=&RID=AYNU7BGD016&SHOW_LINKOUT=yes&SHOW_OVERVIEW=yes&STEP_NUMBER=&WORD_SIZE=11&DISPLAY_SORT=3&HSP_SORT=3#sort_mark)** |  | | --- | --- | --- | --- | --- | --- | --- | --- | | [AE004439.1](http://www.ncbi.nlm.nih.gov/nucleotide/13400023?report=genbank&log$=nucltop&blast_rank=1&RID=AYNU7BGD016) | Pasteurella multocida subsp. multocida str. Pm70, complete genome | [158](http://blast.ncbi.nlm.nih.gov/Blast.cgi#13400023) | 817 | 98% | 3e-35 | 89% |  | | [FM999788.1](http://www.ncbi.nlm.nih.gov/nucleotide/261391559?report=genbank&log$=nucltop&blast_rank=2&RID=AYNU7BGD016) | Neisseria meningitidis 8013, complete genome | [98.7](http://blast.ncbi.nlm.nih.gov/Blast.cgi#261391559) | 352 | 98% | 2e-17 | 85% |  | |
|  |
| | Top of Form  Bottom of Form | Top of Form  Bottom of Form | Top of Form  Bottom of Form | Top of Form  Bottom of Form | | --- | --- | --- | --- | |
| **CRISPR id : tmp_1_Crispr_20** |
| - CRISPR start position : 1925794 ---------- CRISPR end position : 1926093 ---------- CRISPR length : 299 |
| - DR consensus : AGAATAGACTCCCTGATTGCAAAGGGATTCGGATAC |
| - DR length : 36 Number of spacers : 4 |
|  |
| | Top of Form  Bottom of Form | Top of Form  Bottom of Form | Top of Form  Bottom of Form | Top of Form  Bottom of Form | | --- | --- | --- | --- | |

***Additional file 1: Table S5.*** *Bidirectional BLASTp hits for all genes with greater than 90% identity between DSM 24701 and three of the closest fully sequenced genomes, B. avium, Taylorella equigenitalis and T. asigenitalis*

| **Contig** | **Gene** | **Length** | **function** |
| --- | --- | --- | --- |
| **10** | 109 | 123 | LSU ribosomal protein L14p (L23e) |
| **15** | 268 | 45 | LSU ribosomal protein L34p |
| **27** | 556 | 73 | Translation initiation factor 1 |
| **32** | 690 | 139 | LSU ribosomal protein L16p (L10e) |
| **32** | 693 | 92 | SSU ribosomal protein S19p (S15e) |
| **39** | 945 | 78 | SSU ribosomal protein S12p (S23e) |
| **39** | 946 | 157 | SSU ribosomal protein S7p (S5e) |
| **65** | 1447 | 144 | LSU ribosomal protein L11p (L12e) |
| **72** | 1540 | 104 | SSU ribosomal protein S10p (S20e) |
| **75** | 1565 | 74 | ATP synthase C chain (EC 3.6.3.14) |
| **75** | 1570 | 467 | ATP synthase beta chain (EC 3.6.3.14) |
| **77** | 1647 | 83 | RNA-binding protein Hfq |

**Additional file 1: Table S6.** A subset of the 302 predicted proteins from RAST annotation that are unique to DSM 24701 in a blast comparison of DSM 24701, *B. avium, T. equigenetalis and T. asigenitalis.* 179 of the 302 unique genes were annotated as hypothetical proteins, without a predicted function.

| **Contig** | **Gene** | **Length** | | **function** |
| --- | --- | --- | --- | --- |
| **1** | 9 | 262 | COG3306: Glycosyltransferase involved in LPS biosynthesis | |
| **2** | 51 | 87 | Prevent-host-death protein | |
| **2** | 39 | 127 | Error-prone repair protein UmuD (EC 3.4.21.-) | |
| **4** | 58 | 340 | transposase | |
| **11** | 151 | 184 | COG3306: Glycosyltransferase involved in LPS biosynthesis | |
| **13** | 215 | 459 | COG2027: D-alanyl-D-alanine carboxypeptidase (penicillin-binding protein 4)( EC:3.4.16.4 ) | |
| **18** | 386 | 255 | DNA replication protein dnaC | |
| **23** | 470 | 55 | CRISPR-associated protein Cas2 | |
| **24** | 525 | 408 | Nitrate/nitrite transporter | |
| **24** | 526 | 461 | Nitrate/nitrite transporter | |
| **31** | 631 | 746 | Nitric-oxide reductase (EC 1.7.99.7), quinol-dependent | |
| **32** | 717 | 289 | Metallo-beta-lactamase superfamily protein PA0057 | |
| **32** | 719 | 85 | Transcriptional regulator, HxlR family | |
| **33** | 749 | 910 | type III restriction enzyme | |
| **33** | 748 | 669 | Type III restriction-modification system methylation subunit (EC 2.1.1.72) | |
| **34** | 807 | 303 | CRISPR-associated protein, Cas6 | |
| **34** | 802 | 625 | CRISPR-associated protein, Csm1 family | |
| **34** | 804 | 237 | CRISPR-associated RAMP Csm3 | |
| **34** | 805 | 319 | CRISPR-associated RAMP protein, Csm4 family | |
| **34** | 806 | 540 | CRISPR-associated RAMP protein, Csm5 family protein | |
| **36** | 873 | 173 | tight adherence protein E | |
| **36** | 868 | 378 | Type II/IV secretion system ATPase TadZ/CpaE, associated with Flp pilus assembly | |
| **36** | 865 | 150 | Type IV prepilin peptidase TadV/CpaA | |
| **36** | 872 | 230 | TadD | |
| **39** | 944 | 403 | Tryptophan-specific transport protein | |
| **52** | 1246 | 204 | phage-related exonuclease | |
| **52** | 1248 | 299 | Phage-related protein | |
| **54** | 1280 | 303 | CRISPR-associated protein Cas1 | |
| **54** | 1281 | 109 | CRISPR-associated protein Cas2 | |
| **54** | 1279 | 999 | CRISPR-associated protein, Csn1 family | |
| **60** | 1388 | 302 | UDP-N-acetylglucosamine 2-epimerase (EC 5.1.3.14) | |
| **60** | 1357 | 158 | Nitric oxide-dependent regulator DnrN or NorA | |
| **60** | 1355 | 476 | conserved hypothetical protein; possible mucoidy inhibitor-related protein | |
| **66** | 1464 | 680 | Protein TadG, associated with Flp pilus assembly | |
| **67** | 1501 | 323 | Arsenical pump-driving ATPase (EC 3.6.3.16) | |
| **75** | 1587 | 149 | Transcriptional regulator, MarR family | |
| **75** | 1592 | 779 | type I restriction-modification system, M subunit, putative | |
