# Supplementary figures and images for: A genomic perspective on a new bacterial genus and species from the Alcaligenaceae family, Basilea psittacipulmonis
Source: BMC Genomics. 2014 Mar 1;15:169. doi: 10.1186/1471-2164-15-169 (PMC4028982; doi:10.1186/1471-2164-15-169)

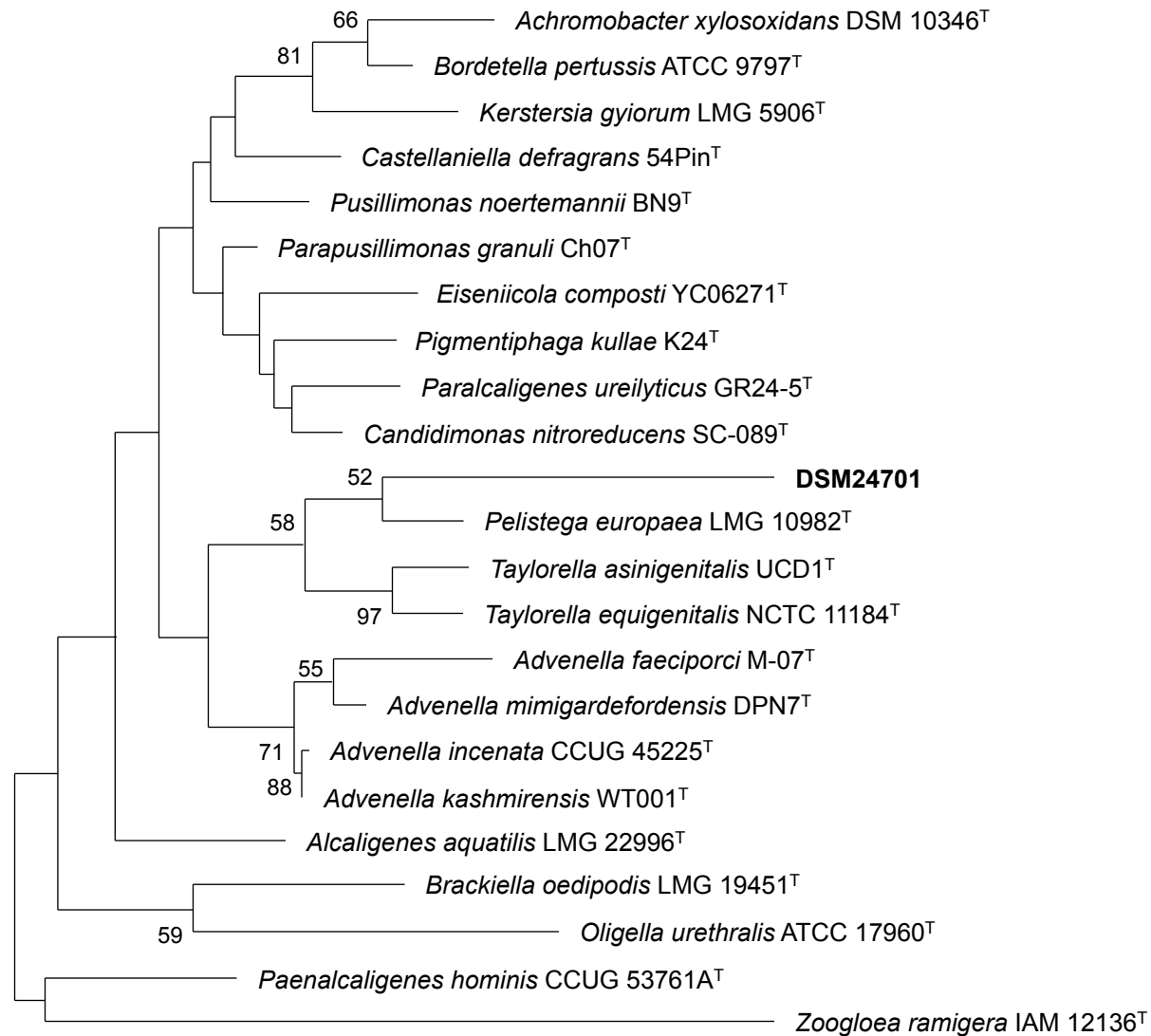

ML

0.01

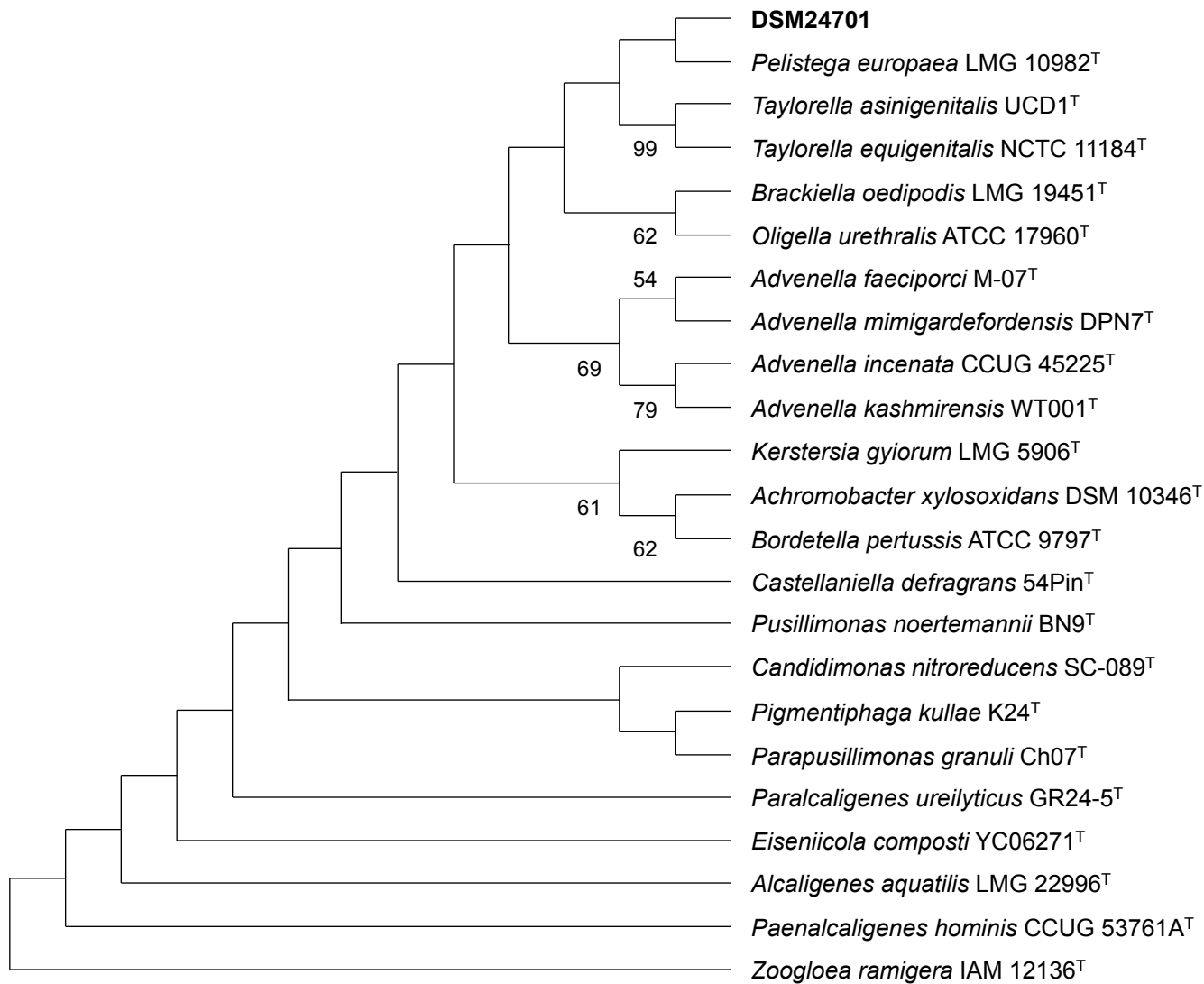

MP

Supplement: Additional file 2: Figure S1 — Phylogenetic trees based on maximum-likelihood (A) and maximum-parsimony (B) analyses of the rRNA gene sequences showing the relationships of DSM 24701 with type species of the family Alcaligenaceae. Type strains of the genera Advenella and Taylorella were also included and the sequence of Zoogloea ramigera IAM 12136 was used as an outgroup. Bootstrap values greater than 50% based on 1000 replications are indicated at branching nodes. Bar, 0.01 substitution per nucleotide position. [file 1471-2164-15-169-S2.PDF]

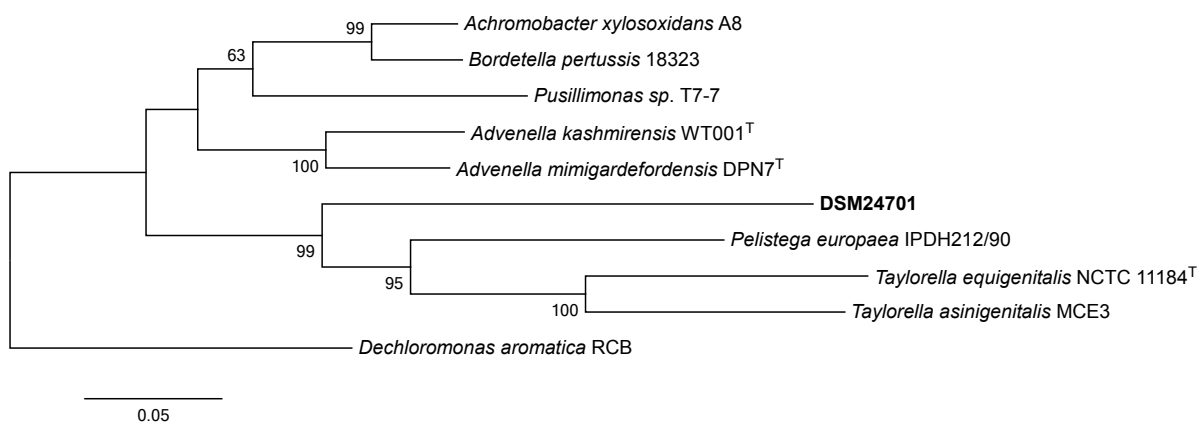

Supplement: Additional file 3: Figure S2 — Phylogenetic tree inferred from rpoB gene sequence comparison showing the relationships of DSM24701 with selected members of the family Alcaligenaceae. All type species of the genera within the family Alcaligenaceae, for which the rpoB sequences were available, were included. For the genera Advenella and Pusillimonas, non-type species were included. The genus Taylorella was represented by the type species (T. equigenitalis) and a non-type species (T. asinigenitalis). Whenever possible, the type strains of the species were used. The sequence of Dechloromonas aromatica RCB was used as an outgroup. The tree was constructed by using the neighbour-joining method. Bootstrap values greater than 50% based on 1000 replications are indicated at branching nodes. Bar, 0.05 s. [file 1471-2164-15-169-S3.PDF]

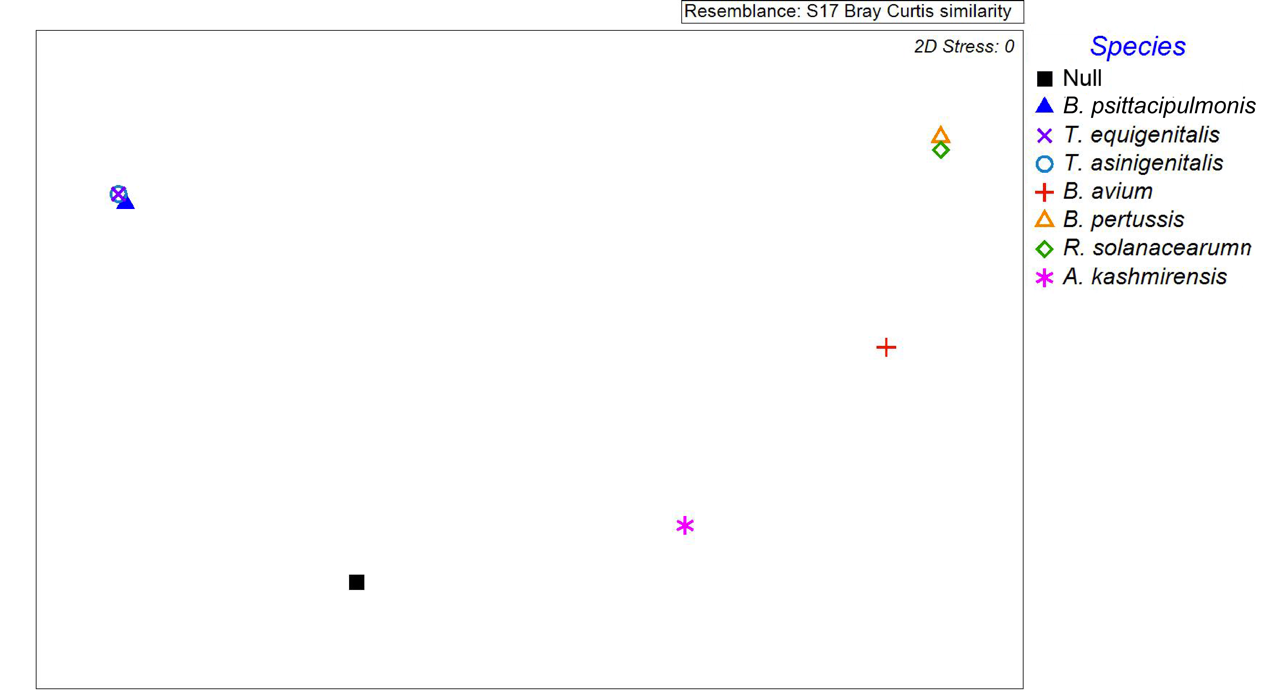

Supplement: Additional file 4: Figure S3 — Dinucleotide usage profile of DSM 24701 and several closely related fully sequenced bacteria. Multi-Dimensional scaling of a Bray-Curtis distance matrix of dinucleotide abundance tables is shown. [file 1471-2164-15-169-S4.TIFF]

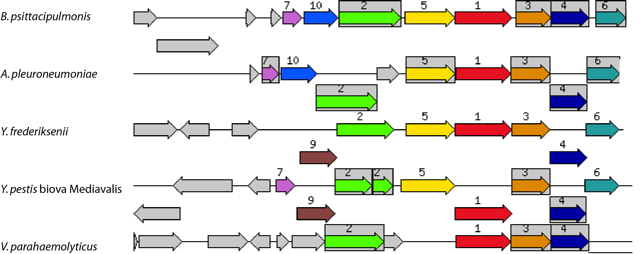

Supplement: Additional file 5: Figure S4 — The tad locus on contig 35 of the DSM 24701. Numbered genes code for: 1. TadA, 2. RcpA, 3. TadB, 4. TadC, 5. TadZ, 6. TadD, 7. TadV, 8. Hypothetical protein, 9. Putative membrane protein, 10. RcpC, 11 Putative membrane protein, 12. TolR. [file 1471-2164-15-169-S5.TIFF]
